# Unraveling the Influence of Structural Complexity, Environmental, and Geographic Factors on Multi‐Trophic Biodiversity in Forested Landscapes

**DOI:** 10.1002/ece3.70907

**Published:** 2025-02-16

**Authors:** Ayanna St. Rose, Kusum Naithani

**Affiliations:** ^1^ Department of Biological Sciences University of Arkansas Fayetteville Arkansas USA

**Keywords:** beetle diversity, bird diversity, canopy structural complexity, combined structural complexity index, LiDAR, maximum height, multi‐trophic diversity index, plant diversity, rugosity, terrain structural complexity

## Abstract

Multi‐trophic diversity is often overlooked in land management decisions due to the absence of cost‐ and time‐effective assessment methods. Here, we introduce a new method to calculate a combined terrain and canopy structural complexity metric using LiDAR data, enabling the prediction of multi‐trophic diversity—a combined diversity metric that integrates diversity across trophic levels. We selected 34 forested sites of the National Ecological Observatory Network to test the model by using observed data on plant presence, beetle pitfall trap, and bird count to calculate multi‐trophic diversity. Our results show that multi‐trophic diversity increases with increasing structural complexity, but this relationship differs across different forest types. The environmental and geographic factors account for about 40% variability in multi‐trophic diversity, which further increases to about 60% when combined with structural complexity. This research offers a powerful approach to evaluate biodiversity at a landscape scale using remotely sensed data and highlights the importance of considering multi‐trophic diversity in land management decisions.

## Graphical abstract

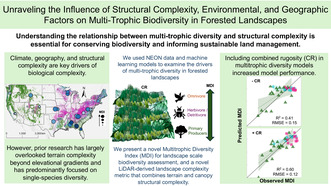

## 1 Introduction

About 1.78 trillion square meter of forested land has been lost globally from 1990 to 2020 (FAO and UNEP 2020). This global decline of forested ecosystems has created an urgency to reevaluate current approaches to forest conservation as forests provide key ecosystem services, including medicinal resources and food supplies, climate change mitigation, opportunities for recreation and cultural enrichment, and habitats for a vast array of terrestrial biodiversity (Angelsen et al. 2014; Houghton 2005; Millennium Ecosystem Assessment (Program) 2005; Neary, Ice, and Jackson 2009). In recent years, there has been a shift among land managers from single‐species conservation (Fleishman, Murphy, and Brussard 2000; Poiani, Merrill, and Chapman 2001; Suter, Graf, and Hess 2002) to multispecies conservation (Barrows et al. 2005; Critchlow et al. 2022). However, progress has been slow primarily due to the lack of cost‐ and time‐effective methods to evaluate it (Suter, Graf, and Hess 2002). Here, we introduce a new model to calculate a combined terrain and canopy structural complexity metric using light detection and ranging (LiDAR) data, improving the prediction of multi‐trophic diversity—a combined diversity metric that integrates diversity across trophic levels.

Recent advancements in LiDAR techniques offer promising prospects for researchers and land managers to investigate structural complexity and biodiversity relationships. Though these products require large processing capacity and storage, and greater variance persists in low‐density LiDAR data, we can still draw great inference from these datasets and variance can be rectified with high‐density data (Zimble et al. 2003; Chow and Hodgson 2009). LiDAR offers a more efficient and less labor‐intensive method of data collection, which is becoming increasingly accessible for natural resource management (Guo et al. 2017; Hardiman et al. 2018; LaRue et al. 2020; Stark et al. 2012; Zellweger et al. 2013; Zimble et al. 2003).

Prior work has highlighted the role of climate (Whittaker, 1971), geography (Hillebrand 2004), and structural complexity (Carrasco et al. 2019; Franklin et al. 1981; Gough et al. 2020; Hardiman et al. 2011; Ishii, Tanabe, and Hiura 2004; Zellweger et al. 2013) in estimating forest age, species richness, and primary productivity. Land management, climate, geography, topography, and environment influence the canopy structural complexity of forested systems (Ehbrecht et al. 2017, 2021; Schall et al. 2018), which generally increases with forest age (Franklin et al. 2002) and can be maintained through sustainable forest management practices (Molina, Marcot, and Lesher 2006). Here, we describe structural complexity as the diversity of the physical attributes and spatial distribution, which can be both vertical and horizontal (Atkins et al. 2018; McElhinny et al. 2005). Vertical complexity refers to the attributes related to the canopy height (e.g., maximum tree height) and terrain (elevation), whereas horizontal complexity refers to the spatial variation in the canopy surface density and layout (Zellweger et al. 2013; Zimble et al. 2003). The role of vertical canopy complexity in supporting species diversity has received greater attention in prior work (de Camargo, Sano, and Vieira 2018; Carrasco et al. 2019; Guo et al. 2017; Müller et al. 2018; Wolf et al. 2012), including the foundational work on niche partitioning (MacArthur and MacArthur 1961). Vertical complexity is positively associated with plant (Gough et al. 2020; Torresani et al. 2020; Walter, Stovall, and Atkins 2021), beetle (Watts and Gibbs 2002), and avian species diversity (Carrasco et al. 2019; Zellweger et al. 2013). In contrast, the role of horizontal complexity (terrain and canopy) in supporting species diversity remains poorly understood and has yielded mixed results. For instance, studies have shown that increasing horizontal complexity positively influenced beetle diversity (Watts and Gibbs 2002) while negatively affecting plant and bird diversity (Gil‐Tena, Saura, and Brotons 2007; Vojík and Boublík 2018). Only a handful of studies have investigated the relationship between terrain structural complexity and biodiversity, and most have focused on elevational gradients (Cisneros et al. 2014; McCain and Grytnes 2010). However, terrain complexity correlates with the spatial distribution of soil nutrients, moisture (Pachepsky, Timlin, and Rawls 2001), and temperature (Frey et al. 2016), which has important implications for aboveground biomass and structure (Zald et al. 2016) and thus can have important implications for multi‐trophic biodiversity.

Previous studies on the effect of structural complexity on species diversity mainly focused on single trophic levels, like avian diversity (Carrasco et al. 2019; Zellweger et al. 2013), and have largely ignored multi‐trophic diversity. Prior work on multi‐trophic diversity employs an additive richness approach (Jones et al. 2022) and the cumulative threshold approach (Allan et al. 2014; Hanusch et al. 2022; Schuldt et al. 2018; Soliveres et al. 2016) that involves calculating diversity based on a threshold for each trophic level (Allan et al. 2014; Schall et al. 2018). The additive approach may cause limitations as the richness of one taxon may be driving observed patterns, and the threshold method poses limitations in instances where there is a lack of data or landscapes have not yet reached the carrying capacity. Here, we introduce two new methods to (1) calculate a combined terrain and canopy structural complexity metric derived from high‐density airborne LiDAR data and (2) estimate a multi‐trophic diversity index that integrates diversity across trophic levels using open data (plant presence, beetle pitfall trap, and bird count) from 34 forested sites of the National Ecological Observatory Network (NEON). Further, we use these new methods to investigate the relative control of environmental, geographic, and structural complexity (terrain and canopy) factors on the multi‐trophic biodiversity at a landscape scale across 34 NEON sites with different forest types. Specifically, we asked the following: (1) *Does a combined terrain and canopy structural complexity index outperform individual metrics in predicting multi‐trophic diversity?* Prior work shows a positive correlation between forest canopy complexity and species diversity (Carrasco et al. 2019; Franklin et al. 1981; Kern et al. 2014; Walter, Stovall, and Atkins 2021). Thus, we expect multi‐trophic diversity to increase with increasing forest canopy structural complexity. Although studies specifically examining the relationship between terrain complexity and multi‐trophic diversity are currently lacking, we hypothesize that multi‐trophic diversity will follow similar trends of decreasing diversity with increasing elevation (Cisneros et al. 2014) and that a higher terrain complexity (increasing variation in elevation) will negatively influence multi‐trophic diversity, under the premise that a smoother surface (lower variation in elevation) would have greater tree cover and diversity. (2) *Does the relationship between structural complexity and multi‐trophic diversity exhibit variations across different forest types?* Given the absence of previous research on the impact of forest type on the complexity–biodiversity relationship, we anticipate that the trend of rising multi‐trophic diversity with increasing structural complexity will remain consistent across various forest types. Nonetheless, slight variations may arise due to differences in their canopy structure. (3) *What is the relative contribution of structural complexity, environmental, and geographic factors in predicting multi‐trophic diversity in forested landscapes?* Prior work showed that using environmental variables and vertical canopy structure resulted in improved predictions of bird diversity than either variable alone (Carrasco et al. 2019). We expect that including environmental and geographical factors in addition to canopy and terrain complexity will improve multi‐trophic diversity predictions.

## 2 Data Products and Data Processing

**FIGURE 1:**
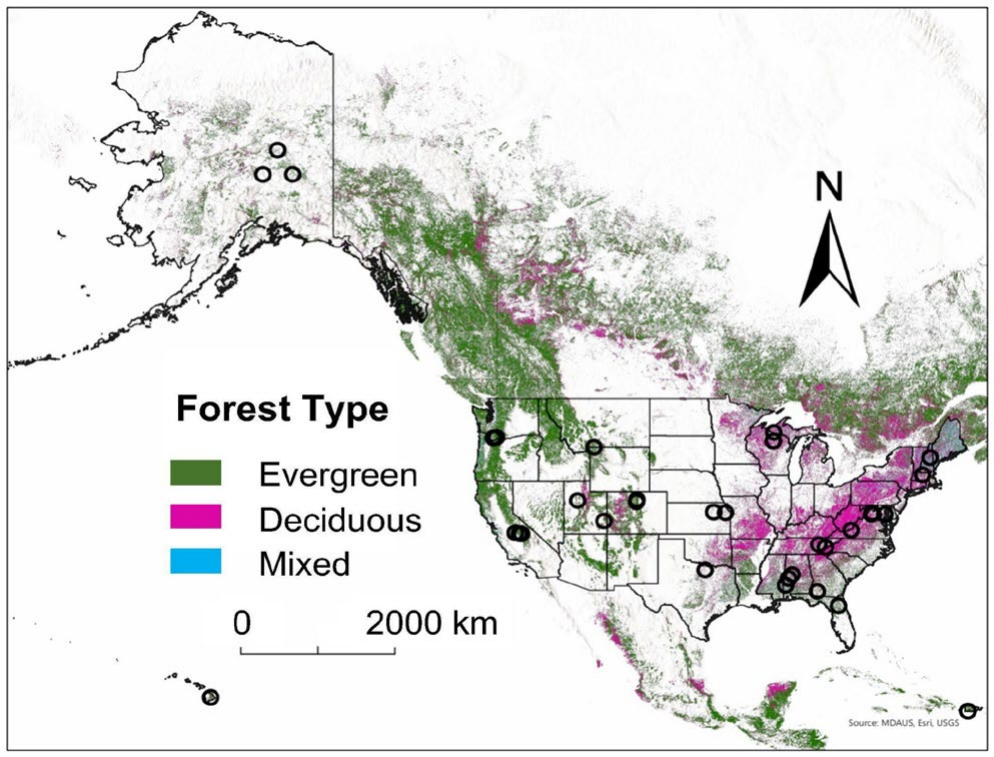
Map of the study area showing locations of 34 forested sites of the National Ecological Observatory Network. Darker circles indicate overlap of sites that appear closer due to the scale of the map. The World Forest data layer in ArcGIS Pro (MDA US BaseVue 2013, ESRI) is used for displaying different forest types (evergreen [dark green], deciduous [pink], and mixed [blue]).

**FIGURE 2:**
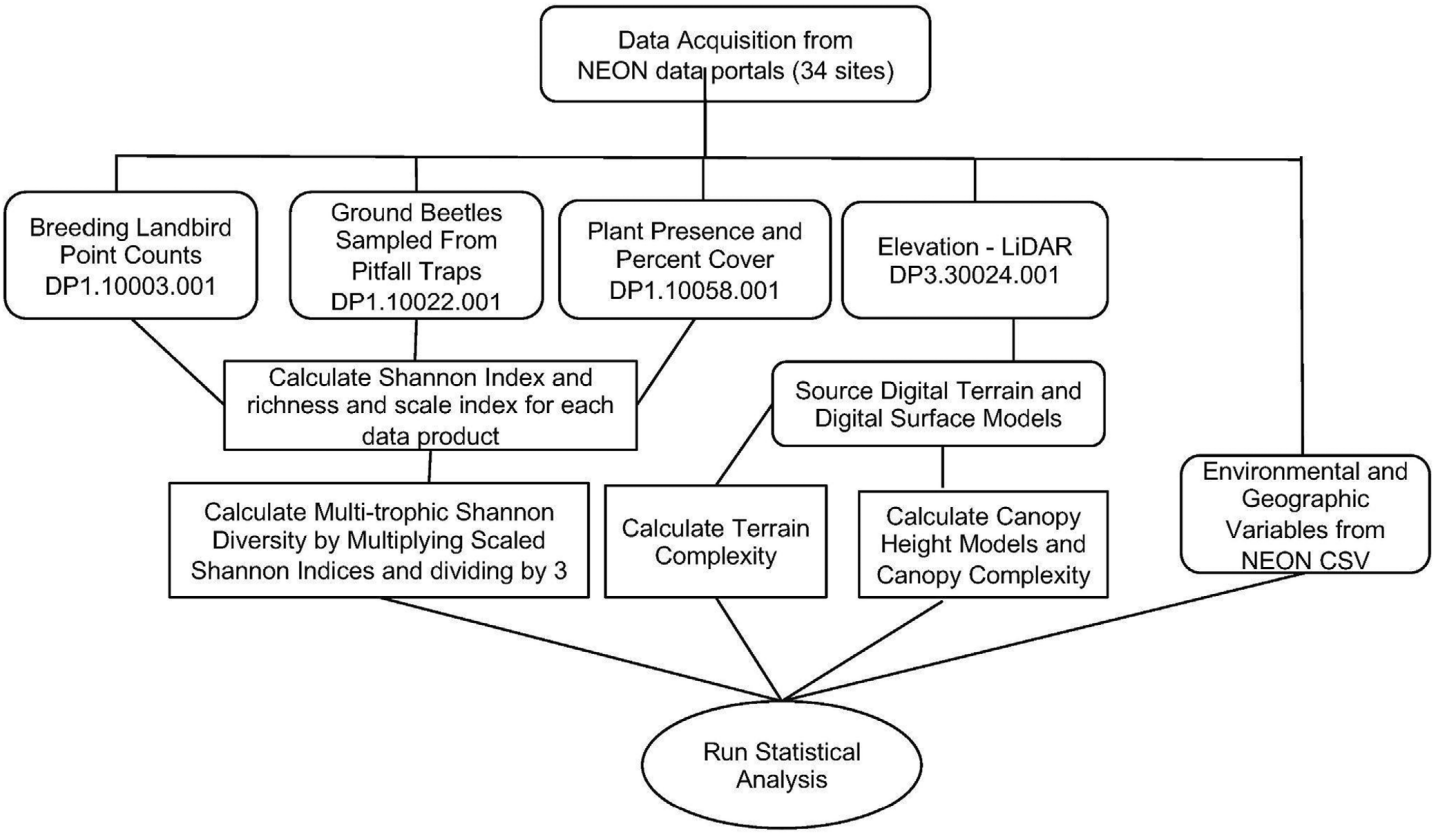
Methodology workflow from data acquisition and data products in the rectangles with rounded edges, and R processing in the rectangles and statistical analysis in the elongated oval.

We selected 34 forested sites (Figure 1 and Table 1) from the NEON for this study. NEON is an open‐source continental‐scale observatory network, which collects, standardizes, and processes airborne remote sensing, observational sampling, and automatic instrument measurements data (Nagy et al. 2021). We used the NEON field site metadata (NEON 2023c) and selected the sites with “forest” National Land Cover Data (NLCD) class keyword in the “nlcd class” column. There were 54 field sites with “forest” listed as part of their NLCD class. Only sites (*n* = 34, Figure 1 and Table 1) with available data for birds, beetles, and plants were selected for further analyses (see Figure 2 for the data analysis workflow).

**TABLE 1:** Site names, site ID, domain ID, state, latitude (Lat), longitude (Lon), mean annual temperature (MAT), mean annual precipitation (MAP), year of the NEON Airborne Observation Platform flight (AOP Flight), forest type (evergreen, deciduous, and mixed forests), and number of species within each trophic level (plant | beetle | bird).

Site	Site ID	Domain ID	State	Lat (°)	Lon (°)	MAT (°C)	MAP (mm)	AOP flight	AOP area (km^2^)	Forest type	Plant | beetle | bird
Abby Road NEON	ABBY	16	WA	45.76244	−122.33032	10.0	2451	2018	132.88	Evergreen	299 | 47 | 83
Bartlett Experimental Forest NEON	BART	1	NH	44.06389	−71.28738	6.2	1325	2022	110.66	Mixed	148 | 26 | 80
Blandy Experimental Farm NEON	BLAN	2	VA	39.03370	−78.04179	12.1	983	2019	93.59	Deciduous	445 | 87 | 83
Caribou‐Poker Creeks Research Watershed	BONA	19	AK	65.15401	−147.50258	−3.0	262	2021	151.62	Evergreen	114 | 16 | 49
Lyndon B. Johnson National Grassland NEON	CLBJ	11	TX	33.40123	−97.57000	17.5	926	2019	129.80	Deciduous	564 | 56 | 133
Delta Junction NEON	DEJU	19	AK	63.88112	−145.75136	−3.0	305	2021	143.20	Evergreen	229 | 29 | 56
Dead Lake NEON	DELA	8	AL	32.54173	−87.80390	17.6	1372	2021	128.63	Evergreen	489 | 26 | 77
Great Smoky Mountains National Park NEON	GRSM	7	TN	35.68896	−83.50195	13.1	1375	2016	140.61	Deciduous	375 | 48 | 86
Guanica Forest NEON	GUAN	4	PR	17.96955	−66.86870	23.0	840	2018	108.46	Evergreen	311 | 5 | 78
Harvard Forest and Quabbin Watershed NEON	HARV	1	MA	42.53691	−72.17265	7.4	1199	2022	152.10	Mixed	457 | 37 | 110
Healy NEON	HEAL	19	AK	63.87580	−149.21335	−1.3	385	2021	112.11	Evergreen	175 | 25 | 41
The Jones Center at Ichauway NEON	JERC	3	GA	31.19484	−84.46862	19.2	1308	2019	141.00	Mixed	1137 | 43 | 86
Konza Prairie Biological Station NEON	KONZ	6	KS	39.10077	−96.56308	12.4	870	2020	129.22	Deciduous	487 | 57 | 119
Lenoir Landing NEON	LENO	8	AL	31.85386	−88.16118	18.1	1386	2021	99.75	Deciduous	383 | 36 | 74
Mountain Lake Biological Station NEON	MLBS	7	VA	37.37831	−80.52485	8.8	1227	2018	87.40	Deciduous	291 | 47 | 81
Moab NEON	MOAB	13	UT	38.24828	−109.38827	10.1	319	2019	98.44	Evergreen	213 | 17 | 85
Niwot Ridge NEON	NIWO	13	CO	40.05425	−105.58237	0.3	1005	2020	90.00	Evergreen	316 | 43 | 54
Onaqui NEON	ONAQ	15	UT	40.17760	−112.45245	9.0	288	2022	131.30	Evergreen	250 | 16 | 82
Oak Ridge National Lab NEON	ORNL	7	TN	35.96000	−84.28000	18.1	1386	2018	72.00	Deciduous	505 | 83 | 115
Ordway‐Swisher Biological Station NEON	OSBS	3	FL	29.68928	−81.99343	20.9	1302	2019	78.50	Evergreen	723 | 43 | 82
Pu'u Maka'ala Natural Area Reserve NEON	PUUM	20	HI	19.55309	−155.31731	12.7	2657	2020	129.80	Evergreen	178 | 14 | 12
Rocky Mountains NEON	RMNP	10	CO	40.27590	−105.54596	2.9	731	2022	137.70	Evergreen	315 | 34 | 71
Smithsonian Conservation Biology Institute NEON	SCBI	2	VA	38.89293	−78.13949	11.6	1126	2019	90.00	Deciduous	565 | 78 | 90
Smithsonian Environmental Research Center NEON	SERC	2	MD	38.89013	−76.56001	13.6	1075	2019	84.15	Deciduous	421 | 76 | 110
San Joaquin Experimental Range NEON	SJER	17	CA	37.10878	−119.73228	16.4	540	2021	111.00	Evergreen	300 | 16 | 116
Soaproot Saddle NEON	SOAP	17	CA	37.03337	−119.26219	13.4	900	2019	85.59	Evergreen	339 | 22 | 81
Steigerwaldt‐Chequamegon NEON	STEI	5	WI	45.50894	−89.58637	4.8	797	2016	103.00	Mixed	620 | 46 | 103
Talladega National Forest NEON	TALL	8	AL	32.95047	−87.39326	17.2	1383	2021	129.32	Mixed	720 | 54 | 103
Lower Teakettle NEON	TEAK	17	CA	37.00583	−119.00602	8.0	1223	2019	134.90	Evergreen	279 | 14 | 81
Treehaven NEON	TREE	5	WI	45.49369	−89.58571	4.8	797	2022	152.60	Mixed	585 | 57 | 83
University of Kansas Field Station NEON	UKFS	6	KS	39.04043	−95.19215	12.7	990	2020	74.40	Deciduous	545 | 75 | 93
University of Notre Dame Environmental Research Center NEON	UNDE	5	MI	46.23391	−89.53725	4.3	802	2022	142.08	Mixed	554 | 38 | 123
Wind River Experimental Forest NEON	WREF	16	WA	45.82049	−121.95191	9.2	2225	2019	122.122	Evergreen	160 | 15 | 65
Yellowstone National Park NEON	YELL	12	WY	44.95348	−110.53914	3.4	493	2020	111.38	Evergreen	500 | 43 | 105

### 2.1 NEON Data Products

On February 21, 2023, we downloaded all available data from 2013 to 2022 for breeding landbird point counts (DP1.10003.001), ground beetles sampled from pitfall traps (DP1.10022.001), plant presence, and percent cover (DP1.10058.001) at the site scale for 34 sites across 17 ecoclimatic domains (NEON 2022; 2023a, 2023b, 2023c). The experimental design for collecting observational data is spatially consistent across all sites, making it suitable for landscape scale analysis used in this study. We removed rows containing unknown species denoted by empty rows before calculating species richness, abundance, and diversity indices (Shannon and Simpson) for plants, beetles, and birds at each site using the vegan (v 2.5–7) package (Oksanen et al. 2020) in R (R Core Team 2023). We also downloaded the most recent data for elevation—LiDAR (DP3.30024.001), containing terrain models and surface models, for calculating terrain complexity and canopy structural complexity indices (Figure 2).

### 2.2 Breeding Land Bird (DP1.10003.001)

We selected *brd_countdata* tables containing bird count data and the *brd_perpoint* containing metadata for birds per point. This data product samples small birds that are only associated with terrestrial ecosystems during the first half of the breeding season. The detailed sampling efforts, optimal sampling dates, and spatial and temporal scales are described in the Neon User Guide to Breeding Landbird Point Counts (DP1.10003.001) document (Thibault 2022). Briefly, a point count method within a predetermined 9‐point grid area separated by 250 m, with about 10 grids per site, was used to record birds that were seen and heard within a 6‐min point count. Counts were done once per year for larger plots and twice per year for smaller plots at the same sampling locations, although some point locations may be moved if there was a disturbance event in that area. Location IDs and coordinates ensure proper recording of this instance. Quality checks were performed on all data points to prevent errors or misidentification of birds.

### 2.3 Ground Beetle Sampling From Pitfall Traps (DP1.10022.001)

We selected taxonomic identification (bet_expertTaxonomistIDProcessed), which contains taxonomic data tables and utilized data on the counts and taxa of ground beetles within each site. Please see the detailed description of ground beetle sampling efforts in NEON protocols (LeVan 2022). Briefly, deli containers of the same size with isopropyl ethanol were used as the pitfall traps. Traps were positioned to the east, west, and south of ten 40‐m base plots. Plots were set up several meters from forest edges, roads, and buildings, and 20 m from the center of the NEON site. Data were collected every 2 weeks in the growing season and recorded by day of collection for every year. If possible, the sampling location remains consistent during every year, but some locations may be moved if a disturbance occurs. Sampling efforts remained congruent among sites; efforts were recorded by trap identification and thus can be scaled up for each site.

### 2.4 Plant Presence and Percent Cover (DP1.10058.001)

We selected and merged the *div_1m2Data* table, containing the plant species identifications within 1 m^2^ subplots, and the *div_10m2Data100m2Data* table containing plant species identifications within 10 and 100 m^2^ subplot variables. This combination accounts for the identifiable species in all 400 m^2^ areas in each site. The diversity indices were calculated based on the taxonomic identification column. Please see the NEON Protocols (Barnett 2022) for a detailed description of the sampling efforts, timing, and location. Briefly, plant taxa were identified at least once a year for each site during 1–2‐month sampling periods at peak greenness (or peak flowering and fruiting). The identification follows NEON taxonomic identification, which employs codes from the US Department of Agriculture, Natural Resources Conservation Service PLANTS database. Identification of trees, shrubs, and herbaceous plants, such as forbs and grasses, was conducted in 400 m^2^ square plots, which contained nested subplots of 100, 20, 10, and 1 m^2^ (Barnett 2022, 66). There was no resampling of surveyed areas to eliminate circular data analysis.

### 2.5 Elevation LiDAR (DP3.30024.001)

**FIGURE 3:**
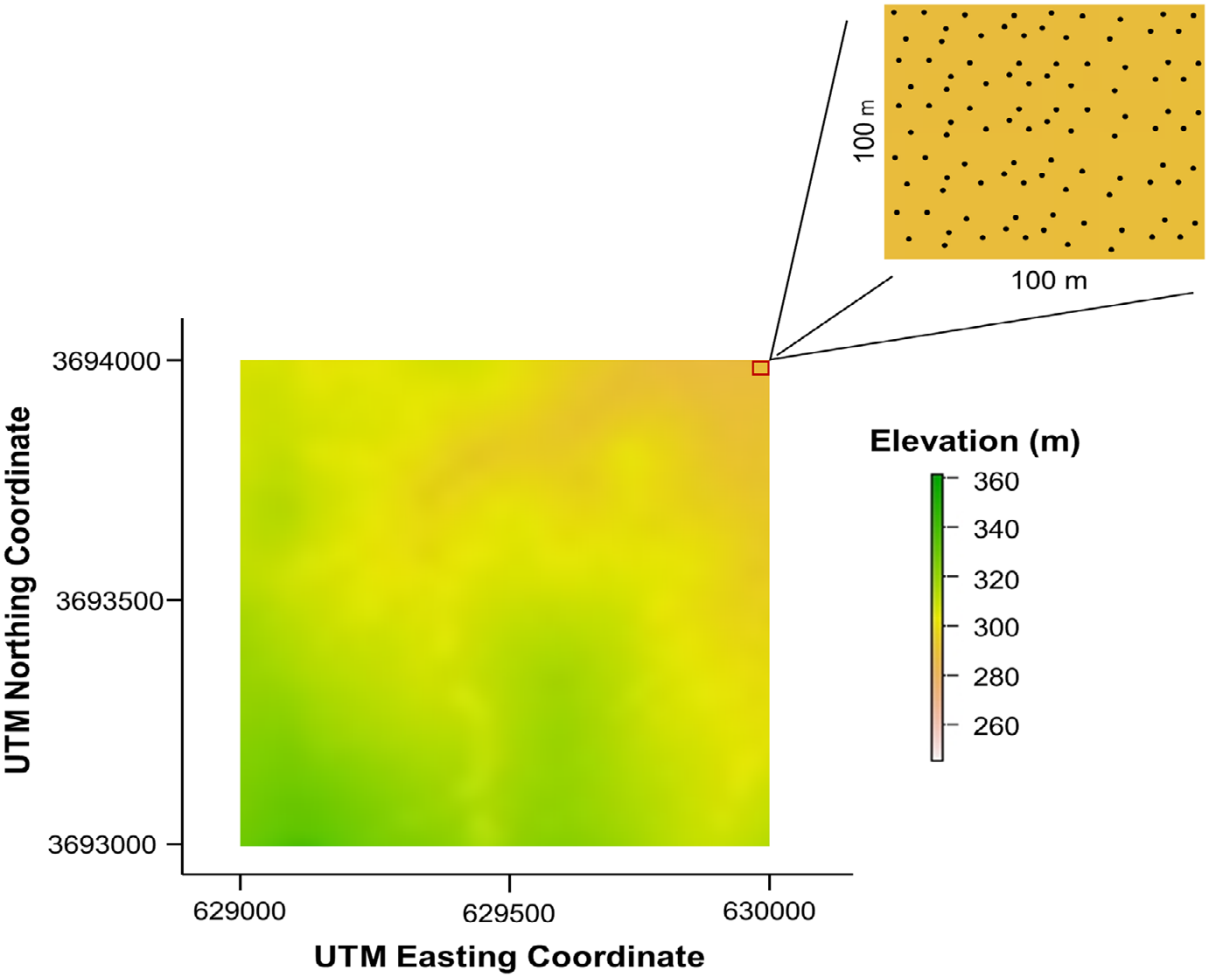
A theoretical depiction of a single pixel within a 1000 m^2^ area clipped from the Digital Terrain Model for the Abby Road NEON site. This 100 m^2^ pixel is highlighted in red, and its value is determined by averaging all the values for a given metric contained within it to represent the overall value for that specific area.

We calculated terrain complexity metrics from the Digital Terrain Model (DTM) rasters that were available for each NEON site; the LiDAR flights were conducted at peak greenness at each site with a vertical resolution of 1.5 m and a spatial resolution of about 0.5 m (Krause and Goulden 2022). We subtracted the raster of the DTM from the Digital Surface Models (DSMs) to create a Canopy Height Model (CHM). We filtered plants below 0.5 m for CHMs to ensure we included shorter trees and understory. When creating rasters for each site, we adjusted the pixel (grid) sizes (1, 5, 10, 15, 20, 25, 50, 70, or 100 m^2^) to get varying within‐site pixel sizes for comparison of structural complexity index (Figure 3).

## 3 Methodology

### 3.1 Multi‐Trophic Diversity Index

**FIGURE 4:**
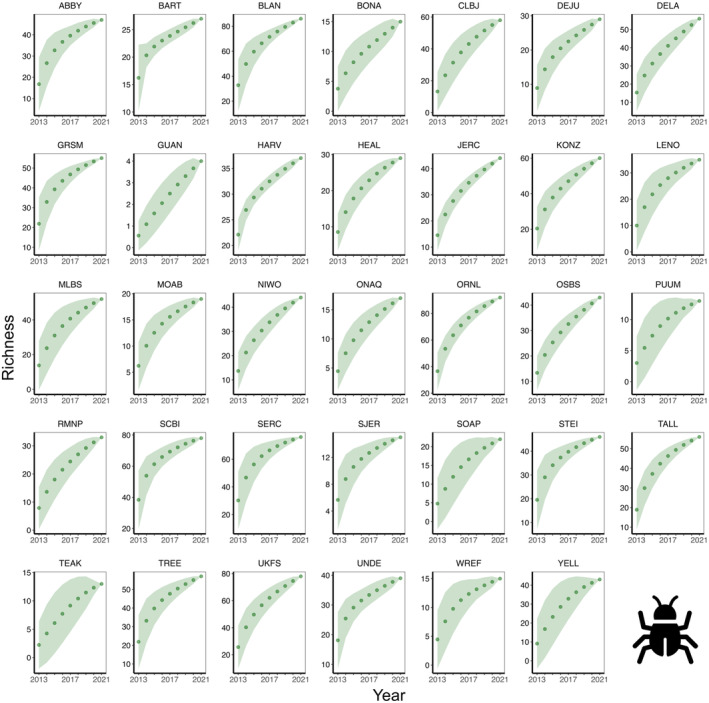
Beetle **s**pecies accumulation curve across 34 NEON forested sites from 2013 to 2021.

**FIGURE 5:**
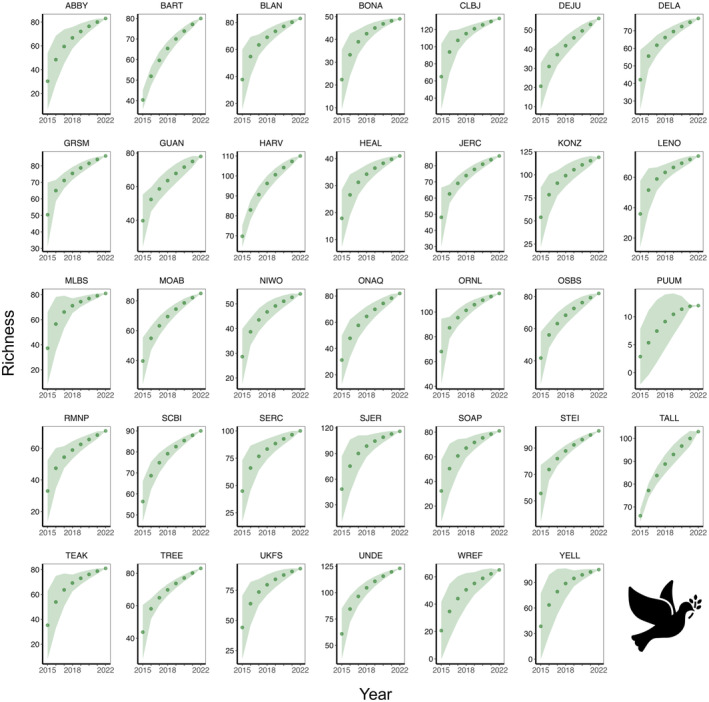
Bird species accumulation curve across 34 NEON forested sites from 2015 to 2022.

**FIGURE 6:**
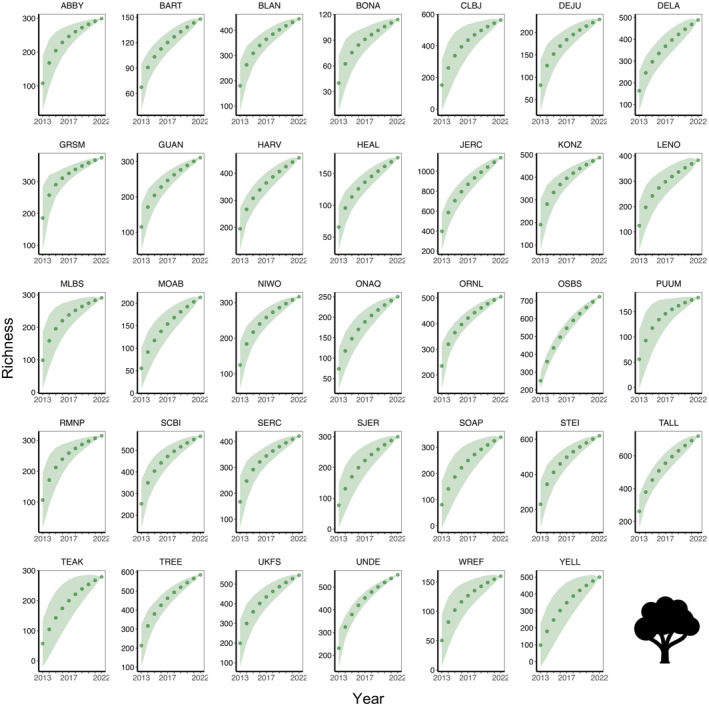
Plant species accumulation curve across 34 NEON forested sites from 2013 to 2022.

**FIGURE 7:**
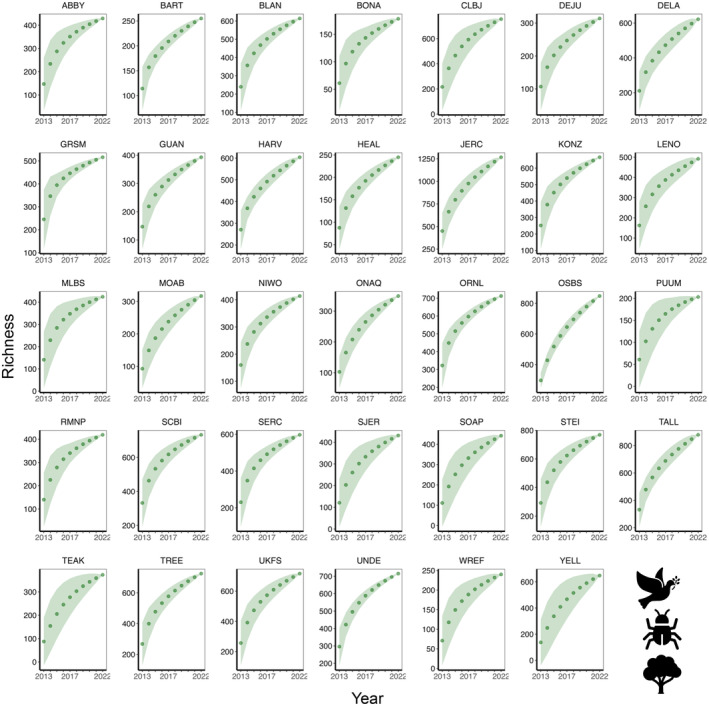
Multi‐trophic species accumulation curve that integrates beetle, bird, and plant species across 34 NEON forested sites from 2013 to 2022.

The site‐specific species accumulation curves indicated that some sites had not reached their maximum potential for diversity (Figures 4, 5, 6, 7), so a threshold method (Allan et al. 2014; Schall et al. 2018) was inappropriate for calculating multi‐trophic diversity. To calculate multi‐trophic diversity, we first scaled the Shannon diversity from 0.1 to 1 for plant, beetle, and bird separately, with the highest diversity site assigned a value of 1 and the lowest diversity site a value of 0.1. For each site, we averaged the scaled diversity indices and divided the result by the number of trophic levels being considered (*n* = 3 in this study; plant, beetle, and bird). Scaling allowed us to capture the variation within a trophic level across sites at a landscape scale, whereas averaging the scaled indices allowed us to account for variation across trophic levels (e.g., omnivore [bird], herbivore [beetle], and primary producer [plant] in this analysis). Please note that this analysis is scale dependent, and results may vary with the number of trophic levels within a site and the number of sites across the landscape.

### 3.2 Structural Complexity Metrics

We calculated nine canopy complexity metrics (top rugosity, entropy, 95th percentile height, rumple, mean maximum canopy height, vertical complexity index [VCI], vegetative area index [VAI], deep‐gap fraction, and cover fraction), six terrain structural complexity metrics (mean elevation, max elevation, slope, roughness, topographic ruggedness index, terrain rugosity [TR]), and two combined terrain and canopy complexity metrics (see Table 2 for equations). All structural complexity metric calculations were done for 1, 5, 10, 15, 20, 25, 30, 40, 50, 60, 70, and 100 m^2^ pixel sizes in each NEON forested site to identify the optimal pixel size for estimating multi‐trophic diversity.

**TABLE 2:** Description of structural complexity metric used in this study.

Metric	Description/Formula *i = 1*, *…*, *s*	Reference
95th percentile height	$Q_{0.95}\;\left(h_{i}\right)$	Bortolot and Wynne (2005)
Mean maximum (outer) canopy height	$\text{MOCH}=\frac{\max\left(h_{i}\right)}{s}$	Atkins et al. (2018)
Canopy rugosity	$\mathrm{CR}=\sqrt{\frac{\Sigma \;{\left(x_{i}-u_{i}\right)}^{2}}{s}}$	Parker and Russ (2004) and Atkins et al. (2018)
Entropy	$E^{\prime }=-\Sigma_{i=1}^{s}\;p_{i}\;\ln\;p_{i}$	Shannon (1948) and Rocchini et al. (2017)
Vertical complexity index	$\mathrm{VCI}=-\Sigma_{i=1}^{s}\frac{p_{i}\;\ln\;p_{i}}{\ln\left(s\right)}$	van Ewijk, Treitz, and Scott (2011)
Vegetative area index	$\mathrm{VAI}=\Sigma_{\mathrm{dz}}^{s}\;\text{Leaf}\;{\text{Area Density}}_{\mathrm{dz}}$	Bouvier et al. (2015)
Rumple	$\text{Rumple}=\frac{1}{s}\Sigma_{i}^{s}{\left(\frac{\text{Canopy Surface Area}}{\text{Ground Surface Area}}\right)}_{i}$	Parker et al. (2004)
Deep‐gap fraction	$\mathrm{DGF}=\left(\frac{\text{Number of pixels with}\;0\;\text{canopy height values}}{s}\right)$	Zhao et al. (2012)
Cover fraction	$\mathrm{CF}=1-\left(\frac{\text{Number of pixels with}\;0\;\text{canopy height values}}{s}\right)$	Zhao et al. (2012)
Terrain ruggedness index	$\mathrm{TRI}=\left|\left(e_{i}-e_{8}\right)\right|$	Wilson et al. (2007), Amatulli et al. (2018), and Riley, DeGloria, and Elliot (1999)
Slope	$s=\tan^{-1}\sqrt{{e_{i}}^{2}+{e_{8}}^{2}}$	Wilson et al. (2007) and Amatulli et al. (2018))
Terrain rugosity	$\mathrm{TR}=\sqrt{\frac{\Sigma \;{\left(x_{i}-u_{i}\right)}^{2}}{s}}$	Shannon (1948)
Terrain roughness	$\text{Roughness}=\frac{1}{\cos\left(\text{slope}\right)}$	Wilson et al. (2007) and Amatulli et al. (2018)
Max elevation	$E=\max\;\left(e_{i}\right)$	Amatulli et al. (2018)
Mean elevation	$\mathrm{me}=\left(\frac{e_{i}}{s}\right)$	Amatulli et al. (2018)
Combined rugosity	$\mathrm{RuC}=\mathrm{CR}\times \frac{1}{\mathrm{TR}}$ Scaled	This paper
Combined roughness	$\mathrm{RoC}=\mathrm{CR}\times \frac{1}{\mathrm{TR}}\times h_{i}$ Scaled	This paper
Multi‐trophic diversity index	$\mathrm{MDI}=\frac{\sum_{j=1}^{n}I\prime_{j}}{n}$	This paper

*Note:* *i* denotes the individual pixel (*i* = 1, …, *s*) and *s* represents the total number of pixels within a forested site. For canopy structural complexity metrics: *h*_*i*_ = maximum canopy height within pixel *i*, *Q*_0.95_ refers to the 95th percentile, *p*_*i*_ = relative abundance of height returns in pixel *i*, *dz* = vertical slices of 1‐m canopy height, *x*_*i*_ = max value (height or elevation) in a pixel *i*, *μ*_*i*_ = mean value (height or elevation) in a pixel *i*, and *n* is the number of trophic levels. For terrain complexity metrics: *e*_*i*_ = maximum elevation within pixel *i* and *e*_8_ = elevation of eight neighboring cells. For multi‐trophic diversity: *I′* is a scaled Shannon diversity index.

Top rugosity or roughness due to variation in the heights was calculated as the standard deviation of the height values within the CHM (Atkins et al. 2018; Parker and Russ 2004). Ninety‐fifth percentile height was calculated for the CHM of each site instead of maximum canopy height to filter out outliers, and the mean maximum canopy height was calculated by calculating the mean of canopy heights within each pixel of the CHM (Atkins et al. 2018; Bortolot and Wynne 2005). Entropy, a diversity and evenness measure across every 1 m vertical slice of the CHM, was calculated by following the Shannon diversity index (Shannon 1948) approach to yield a normalized canopy Shannon diversity index of the height profiles in the CHM that describes the distribution of heights within the forest (de Almeida et al. 2020). VCI is the normalized entropy measure (van Ewijk, Treitz, and Scott 2011). Rumple, a ratio of the outer canopy surface to the ground area (1 km^2^ plot) of the CHM (Jenness 2004; Parker et al. 2004), is a metric that is closely related to canopy closure and was calculated using the lidR package from the CHM raster (Roussel et al. 2020). VAI quantifies the density of vegetation within the canopy at 1 m vertical slices of the CHM (Bouvier et al. 2015). Deep‐gap fraction is calculated by counting the number of pixels with no return value (ground) relative to the entire plot, and cover fraction (1‐ deep‐gap fraction) is an estimate of the canopy cover of the plot (Zhao et al. 2012).

Terrain complexity metrics were calculated from the DTMs. The mean elevation was calculated by averaging the mean elevation for each pixel, and max elevation was calculated by averaging the maximum heights across all pixels within the DTMs. TR was calculated as the standard deviation of the elevation values within the DTM and is a roughness measure that describes the variation in the elevation. Terrain ruggedness index (TRI) describes the local ruggedness of the DTM by taking the absolute value of the mean difference between a given pixel and the surrounding eight pixels (Amatulli et al. 2018; Riley, DeGloria, and Elliot 1999; Wilson et al. 2007). Terrain roughness describes the mean difference between max and min values between a given pixel and the surrounding eight pixels (Amatulli et al. 2018; Wilson et al. 2007). Slope was calculated as the mean slope about a centroid, with eight neighboring cells (Wilson et al. 2007; Amatulli et al. 2018).

We developed two new methods of combined structural complexity indices using scaled rugosity and height metrics. For the first combined terrain and canopy roughness metric, we multiplied the scaled canopy rugosity (CR) to the reciprocal of the scaled terrain rugosity (TR) metric, termed combined rugosity ($\mathrm{RuC}={\mathrm{CR}}_{\text{Scaled}}\times \left[1/\mathrm{TR}\right]$; Table 2).

We used the reciprocal of the terrain complexity because prior work suggests that increased terrain complexity is related to reduced biological complexity (Acharya et al. 2011; Wambugu et al. 2024). The second metric, combined roughness ($\mathrm{RoC}={\mathrm{CR}}_{\text{Scaled}}\times h_{\text{Scaled}}\times \left[1/\mathrm{TR}\right]$; Table 2), multiplies the scaled 95th percentile canopy height value to the first combined metric as height has been shown to be an important metric in biodiversity and structure studies (Carrasco et al. 2019; MacArthur and MacArthur 1961; Zellweger et al. 2013).

### 3.3 Variable Selection

We performed a correlation analysis using Spearman's rho coefficient (*r*_s_) on all structural complexity metrics and the multi‐trophic diversity. We did not expect a linear relationship (Hanusch et al. 2022), so we selected a nonparametric approach to assess the level of association between the diversity indices and the other variables prior to conducting further analysis. The objectives of this analysis were to (1) determine the optimal within‐site pixel size for each metric by selecting the metric that exhibited the highest correlation with the multi‐trophic diversity, considering only statistically significant correlations (*p* ≤ 0.05); (2) investigate the correlation between the metrics and multi‐trophic diversity, giving preference to the metric showing a stronger correlation with the multi‐trophic diversity; and (3) select metrics that exhibited low correlation (below |0.7|) with each other to mitigate the effects of multicollinearity in subsequent analyses (Dormann et al. 2013; LaRue et al. 2020).

### 3.4 Identifying Predictors of Multi‐Trophic Diversity

After selecting the metrics at the optimum pixel size that were not highly correlated with each other, we used the random forest model to identify the top predictors for multi‐trophic diversity. In addition to the structural metrics, we included climate (mean annual precipitation [MAP], mean annual temperature [MAT]), topographical (mean elevation), and geographical (latitude and longitude) variables in the random forest model. For random forest analysis, we used a tuning function to determine the optimal values for parameters such as *mtry* and *ntree*. Specifically, we executed the random forest model with 600 trees while selecting two predictors at each split. To evaluate the model performance, we employed leave‐one‐out cross‐validation and calculated the root mean square error (RMSE) of predictions.

We further explored the bivariate relationship between multi‐trophic diversity and top predictors from random forest analysis.

### 3.5 Investigating Dissimilarity Between Forest Types

We used a nonmetric multidimensional scaling (NMDS) analysis to visualize the difference in multi‐trophic diversity across different forest types. We calculated species abundance from species count data and explored the relative impact of different environmental and structural variables on species abundance at each trophic level using the ecodist (Goslee and Urban 2007) and vegan (Oksanen et al. 2020) packages in R. We used the Bray–Curtis distance method to estimate the influence of environmental and geographic variables on multi‐trophic species abundance grouped by three forest types: evergreen, mixed, and deciduous. We ran an ANOSIM (analysis of similarities), similar to Fontúrbel and Jiménez (2014), to test the difference in the dissimilarity pattern of the forest types observed in the NMDS (Chapman and Underwood 1999). Additionally, we ran a permutational multivariate analysis of variance PERMANOVA to explore the significance of the differences between forested groups and the combined effect of the explanatory variables.

## 4 Results

### 4.1 Pixel Size for Calculating Structural Complexity Metrics

The correlation analysis resulted in low to moderate correlations between multi‐trophic diversity and various structural complexity metrics. Mean elevation showed the best correlation at 40 m (*r*_s_ = −0.44, *p* < 0.01), maximum elevation had a similar correlation with multi‐trophic diversity at 60 and 100 m resolutions (*r*_s_ = −0.50, *p* < 0.01), and slope (*r*_s_ = −0.34, *p* = 0.03) showed significant correlations at 100 m resolution. TRI at 1 m resolution explained the maximum variance in multi‐trophic diversity (*r*_s_ = −0.33, *p* = 0.04). However, the relationship between multi‐trophic diversity and TR was not affected when the pixel size was adjusted before calculating TR, showing consistent correlations across all pixel sizes (*r*_s_ = −0.52, *p* < 0.01).

Canopy structural complexity metrics were highly correlated with multi‐trophic diversity. Combined rugosity at 50 m resolution (*r*_s_ = 0.69, *p* < 0.05) and combined roughness at 1 m (*r*_s_ = 0.70, *p* < 0.05) were highly correlated with multi‐trophic diversity. At 40 m pixel size, the 95th percentile height (*r*_s_ = 0.49, *p* < 0.01), and at 1 m pixel size, VAI (*r*_s_ = 0.46, *p* < 0.01) and mean canopy height (*r*_s_ = 0.50, *p* < 0.01) showed moderate correlation with multi‐trophic diversity. Rumple (*r*_s_ = 0.35, *p* = 0.02) moderately explained the variation in multi‐trophic diversity at 1 m resolution, whereas entropy (*r*_s_ = 0.64, *p* < 0.01) and CR (*r*_s_ = 0.55, *p* < 0.01) showed significant relationships at 50 m resolution. VCI (*r*_s_ = 0.57, *p* < 0.01), deep‐gap fraction (*r*_s_ = 0.37, *p* = 0.04), and canopy fraction (*r*_s_ = −0.37, *p* = 0.03) showed significant correlations at 100 m resolution. For the following analyses, we selected the metrics that were most correlated with multi‐trophic diversity (*p* < 0.05) and had low correlation with each other (*r*_s_ > 0.7). We selected combined rugosity at 60 m resolution, mean maximum height, rumple at 1 m resolution, 95th percentile height at 40 m resolution, and deep‐gap fraction at 100 m resolution to run further analysis.

### 4.2 Top Predictors of Multi‐Trophic Diversity

**FIGURE 8:**
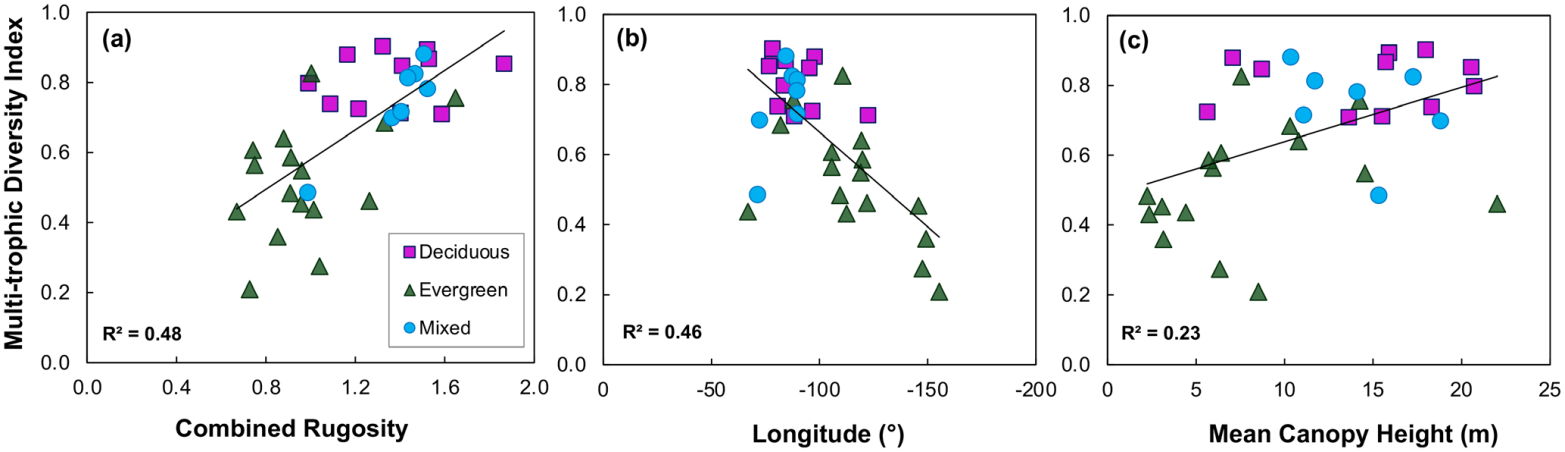
Bivariate relationships between multi‐trophic diversity and the top three predictors of the random forest model: (a) combined rugosity at 60 m resolution, (b) longitude at 1 m resolution, and (c) mean canopy height at 1 m resolution across 34 forested sites of the National Ecological Observatory Network. All models are significant at *p* < 0.05 and represented by the solid line. Different forest types are represented as triangle (evergreen), circle (mixed), and square (deciduous).

The bivariate relationships between multi‐trophic diversity and the top three random forest predictors indicated that combined rugosity accounted for the highest amount of variation in multi‐trophic diversity (48%, *p* < 0.05), closely followed by longitude (46%, *p* < 0.05) and mean canopy height (23%, *p* < 0.05) (Figure 8 and Table 3). Additionally, 95th percentile canopy height, VAI, and MAT were significant independent metrics, explaining 22%, 18%, and 16% of the variation, respectively (*p* < 0.05). Deep‐gap fraction, latitude, rumple, and MAP did not have a significant effect on multi‐trophic diversity (Table 3).

**TABLE 3:** Linear model parameters showing the significance of the relationship between variables and multi‐trophic diversity.

Variable	Deviance explained (%)	Adjusted *R* ^2^	RMSE
Combined rugosity^a^	47.7	0.46	0.14
Longitude^a^	46.4	0.44	0.14
Mean canopy height^a^	22.8	0.20	0.16
95th percentile height^a^	21.9	0.19	0.17
Vegetative area index^a^	18.5	0.16	0.17
MAT^a^	15.8	0.12	0.18
Deep‐gap fraction	9.3	0.07	0.18
Latitude	4.9	0.02	0.19
Rumple	6.7	0.04	0.19
MAP	0.4	−0.03	0.19

^a^ Variables represent significance (*p* < 0.05).

**FIGURE 9:**
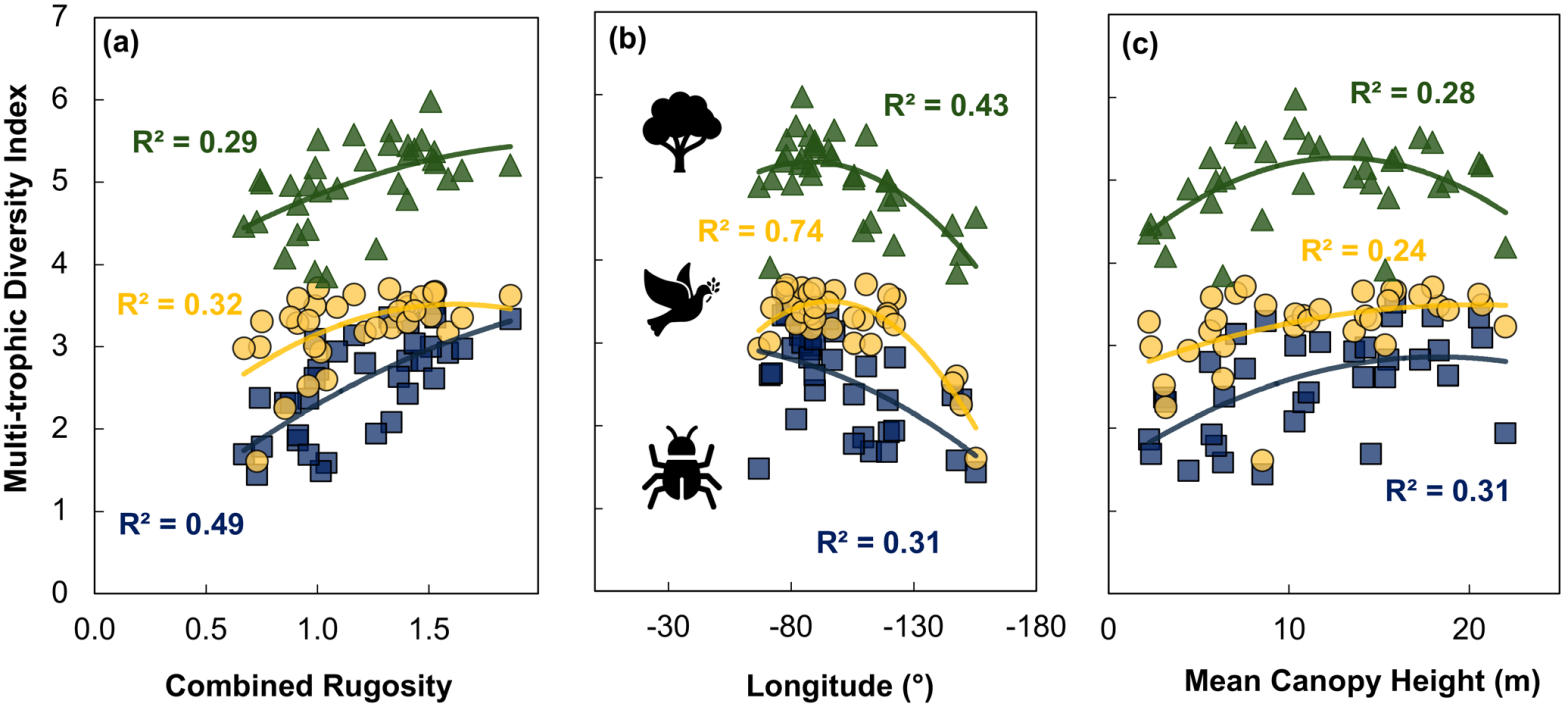
Bivariate relationships between Shannon diversity index of plant (green triangle), bird (yellow circle), and beetle (blue square) and the top three predictors of multi‐trophic diversity from the random forest model: (a) combined rugosity at 60 m resolution, (b) longitude at 1 m resolution, and (c) 95th percentile canopy height at 40 m resolution across 34 forested sites of the National Ecological Observatory Network. All models are significant at *p* < 0.05.

**FIGURE 10:**
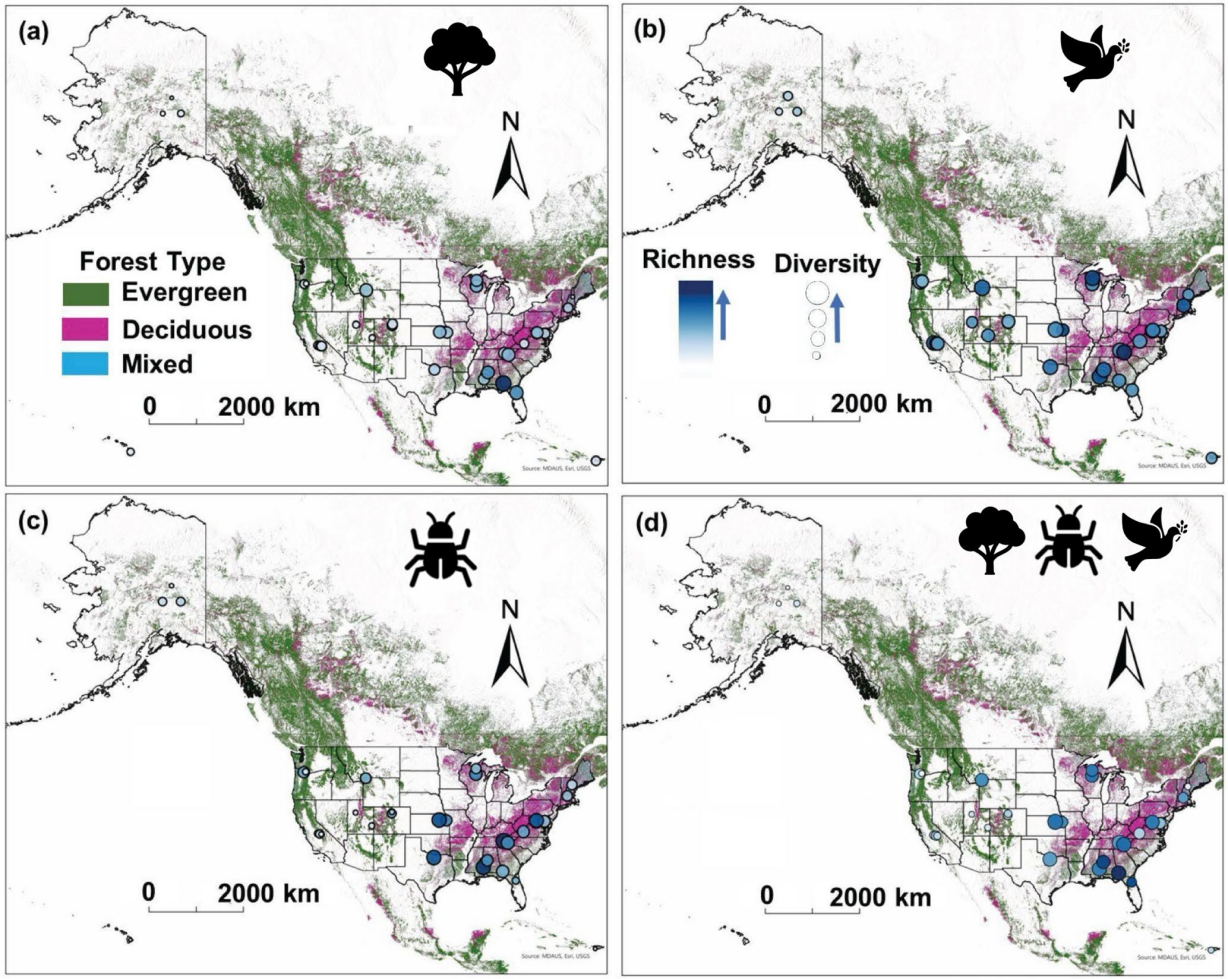
Spatial distribution of species richness and species diversity (Shannon diversity index) of (a) plant, (b) bird, (c) beetle, and (d) multi‐trophic diversity, across 34 forested sites of the National Ecological Observatory Network. The World Forest data layer in ArcGIS Pro (MDA US BaseVue 2013) is used for displaying different forest types (evergreen [dark green], deciduous [pink], and mixed [blue]).

Multi‐trophic diversity increases with combined rugosity (*R* ^2^ = 0.48) and mean canopy height (*R* ^2^ = 0.23), whereas it decreases with decreasing longitude (*R* ^2^ = 0.44) (Figure 8). Generally, mixed and deciduous forests have greater structural complexity (Figure 8a) and mean canopy height (Figure 8c), whereas evergreen forests have a greater gradient of canopy height, combined rugosity, and longitude. The plant, bird, and beetle diversities follow similar trends, increasing with combined rugosity and mean canopy height, and decreasing with changing latitude from east to west (Figure 9). Plant and beetle diversities (and richness) were lower in the western United States, dominated by evergreen forests, and greater in the eastern United States, dominated by deciduous and mixed forests (Figure 10). In contrast, bird diversity increased until mid‐combined rugosity (Figure 9a) and maximum canopy height (Figure 9c), and increased until −100° but decreased further west (Figures 9b and 10b). Multi‐trophic diversity followed similar trends where there was a lower diversity in Hawaii, Alaska, Puerto Rico, and on the West Coast (Figure 10d). In the central United States and around the Appalachian area, there was a higher multi‐trophic diversity and richness (Figure 10d).

### 4.3 Relative Contribution of Geography, Environment, and Complexity in Predicting Multi‐Trophic Diversity

**FIGURE 11:**
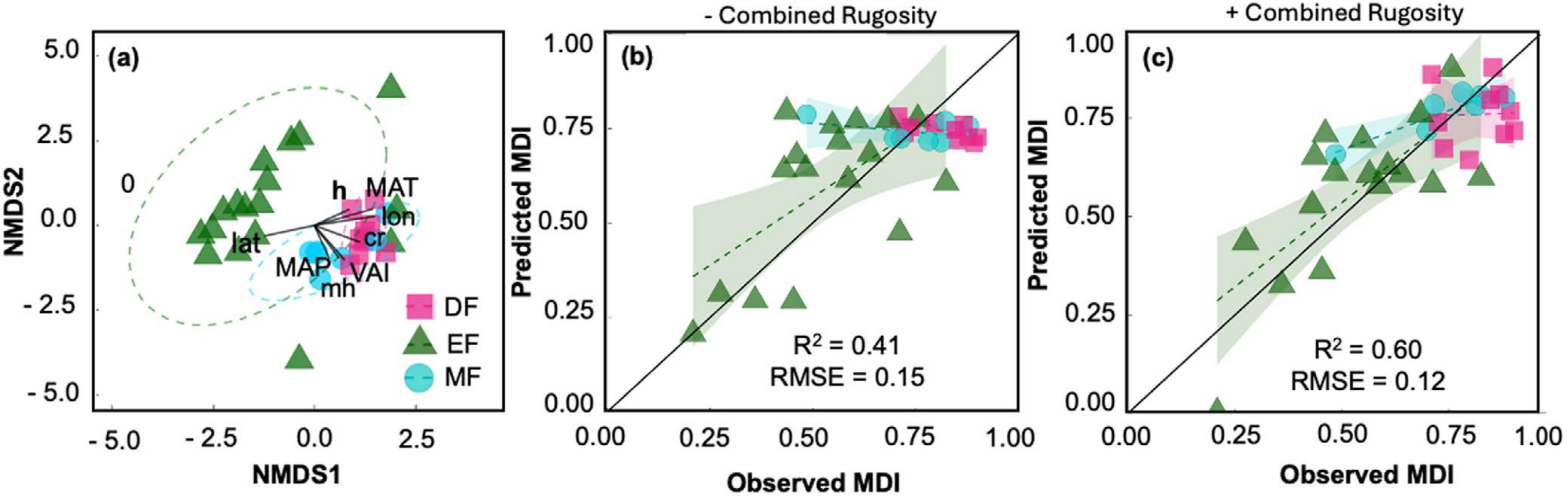
(a) Nonmetric multidimensional scaling (NMDS) plot showing multi‐trophic abundance grouped by forest type across 34 NEON forested sites. The geographical (lat = latitude and lon = longitude (°)), environmental (MAT = mean annual temperature (°C) and MAP = mean annual precipitation (mm)), and structural complexity (mh = mean height (m), cr = combined rugosity, *h* = max canopy height (m), VAI = vegetative area index) variables are shown as vectors. A longer arrow indicates greater influence in the pointed direction. Only significant (*p* < 0.05) variables are shown here. (b) Model goodness‐of‐fit for the polynomial regression model for predicting multi‐trophic diversity (MDI) with geographic and environmental variables (MDI = lon^2^ + MAP^2^ + MAT). (c) The polynomial model with added complexity (MDI = lon^2^ + MAP^2^ + MAT + combined rugosity).

The multi‐trophic diversity within most evergreen forests separated from deciduous and mixed forests, and there was more spread in the evergreen forests (Figure 11a). On the other hand, multi‐trophic diversity in deciduous and mixed forests was grouped together, indicating that these sites are most similar to each other and different from the evergreen sites. Latitude explained about 46% of the variation in the direction of the evergreen forested sites (Figure 11a). The complexity metrics, VAI (*R* ^2^ = 0.46, *p* < 0.05), combined rugosity (*R* ^2^ = 0.44, *p* < 0.05), mean height (*R* ^2^ = 0.38, *p* < 0.05), and 95th percentile height (*R* ^2^ = 0.36, *p* < 0.05), explained the variation in deciduous and mixed forests, along with MAT, longitude (*R* ^2^ = 0.67, *p* < 0.05), and MAP (*R* ^2^ = 0.32, *p* < 0.05). ANOSIM (*R* statistic = 0.23, *p* < 0.05) and PERMANOVA supported that the ranked dissimilarity in species diversity between mixed and deciduous forests was not significantly different (*p* > 0.05), but the dissimilarity of both was significantly different from evergreen forests (Figure 11a). Further analysis using a polynomial regression model, built using geographic and environmental variables, shows that combined rugosity explains an additional 19% variability in MDI when the model was run with and without combined rugosity (Figure 11b,c).

## 5 Discussion

### 5.1 Top Predictors of Multi‐Trophic Diversity

Multi‐trophic and single trophic diversity generally increased with increasing structural complexity (Figures 8a,c and 9a,c), which is consistent with prior work showing that increasing vertical complexity metrics explained most variation in plant (Gough et al. 2020; Torresani et al. 2020; Walter, Stovall, and Atkins 2021), beetle (Watts and Gibbs 2002), and avian diversities (Carrasco et al. 2019; Zellweger et al. 2013). Moreover, a lower tree diversity was characteristic of the rugged terrain of Ecuadorian ridges (Homeier et al. 2010). Previous work on height variation hypothesis suggests that increased variation in the height of the canopy is associated with greater diversity of trees (Torresani et al. 2020) and avian species (Carrasco et al. 2019; MacArthur and MacArthur 1961; Pearson 1971; Yahner 1982; Zellweger et al. 2013) due to increased microhabitats and niche partitioning. Our results extend these findings to multi‐trophic diversity. Combined terrain and canopy complexity explained greater variation in biodiversity than each complexity metric alone, highlighting the effect of terrain complexity on biodiversity.

Overall, vertical complexity variables explained multi‐trophic diversity better than horizontal complexity metrics (Table 3). Variables explaining the horizontal heterogeneity, like deep‐gap fraction and rumple, did not significantly explain the variation in multi‐trophic diversity. Prior work on horizontal complexity effects on bird diversity showed mixed results from weaker (Zellweger et al. 2013) to stronger (Carrasco et al. 2019) relationships in comparison to vertical complexity. Similar patterns have been observed where beetle (Watts and Gibbs 2002) and bat (Erasmy et al. 2021) diversity increased with increasing canopy density, but some studies have shown that plant (Vojík and Boublík 2018) and bird (Gil‐Tena, Saura, and Brotons 2007) diversity decreased with increasing canopy density. The saturation in bird diversity suggests that the maximum species diversity occurs at the mid‐level of vertical complexity (Figure 9), consistent with prior work suggesting that bird diversity showed a similar saturating effect with maximum diversity at the mid‐level complexity (Carrasco et al. 2019).

Longitude played a key role in explaining the variation of multi‐trophic diversity. The sites on the east coast had a higher multi‐trophic diversity than the sites on the western United States (Figures 8 and 9). However, these trends varied for bird diversity, with lower diversity for the Hawaiian and three Alaskan sites (Figures 8 and 9), causing a saturating curve in Figure 5b. Although latitude has been a focal point and identified as a driver of biodiversity in numerous studies (Hillebrand 2004), it did not significantly affect multi‐trophic biodiversity (Table 3) in this study. The differing results may be explained by the smaller latitudinal gradients, as all study sites are within the northern temperate zone.

### 5.2 Relative Contribution of Geography, Environment, and Complexity in Predicting Multi‐Trophic Diversity

The relationships between multi‐trophic diversity and complexity differed in evergreen, deciduous, and mixed forests (Figure 8). Multi‐trophic diversity had the best relationship with the top predictors in mixed forests. We found a significant linear increase in multi‐trophic species diversity with increasing combined structural complexity in mixed forests (Figure 8b). Previous work has shown that mixed forests are more resilient (Pretzsch, Schütze, and Uhl 2013) and thus can sustain high diversity. Additionally, mixed forests are likely to have optimal structural complexity (vertical and horizontal) to support high plant diversity and more diverse food sources (e.g., diverse litter types for beetles, different types of fruits, nuts, and worms for birds) to support a higher diversity of consumers at higher trophic levels.

Prior work integrating environmental and structural complexity metrics is missing or limited to individual species (Gouveia et al. 2014). With the increasing availability of field and remotely sensed data on species diversity, climate, and structure, there is an opportunity to use it for exploratory studies to identify indicators of multi‐trophic biodiversity. Multi‐trophic diversity and structural complexity showed weak or no relationship across evergreen forests, whereas latitude was a significant predictor of multi‐trophic diversity in this type of forest (Figure 8). The lower multi‐trophic diversity in evergreen forests can be explained by lower plant diversity and availability of food for consumers compared to those in deciduous and mixed forests. It is challenging to separate the effects of climate, geography, and structural complexity due to the inherent feedback and interconnected nature of these factors. However, our results show that combined rugosity explains an additional 19% variability in MDI when a polynomial regression model was run with and without (Figure 11) combined rugosity. Our results suggest that structural complexity metrics can be used as indicators of landscape‐scale multi‐trophic diversity.

### 5.3 Limitations

Although LiDAR products save time, money, and resources, these data products are usually very large and require a greater storage and processing power. Therefore, our use of DTM and DSM study plots was suitable, convenient, and efficient. We noticed that the species accumulation curves for bird, beetle, and plant did not plateau within a year (Figures 4, 5, 6), so we used all available data from NEON. As NEON continues to collect more field data on species diversity, this will allow a more robust analysis in future studies to better understand the drivers of multi‐trophic biodiversity at a continental scale. Although our research shows that openly available data with larger resolution can be used to quantify combined complexity, it is important to note that the strength and direction of the relationships between multi‐trophic diversity and complexity can vary by resolution or grain size. For example, deep‐gap fraction had a weak negative relationship with diversity at 1 m (*r*_s_ = −0.17) but a stronger positive relationship at 100 m (*r*_s_ = 0.37). The issue of scale and resolution size is not new to ecology (Levin 1992), and exploring how these relationships vary at multiple scales is important to inform better analyses and management plans.

## 6 Conclusions

Our study examines how terrain and canopy structural complexity, along with environmental and geographical factors, influences multi‐trophic diversity across the continental United States. We found that multi‐trophic diversity increases with greater structural complexity, with mixed and deciduous forests exhibiting higher structural complexity and mean canopy height, whereas evergreen forests being less structurally complex. However, evergreen forests display a greater gradient of canopy height, combined rugosity, and longitude. Geography and environment account for about 40% of the variability in multi‐trophic diversity across forested landscapes, which increases to about 60% with the addition of combined structural complexity. Further investigation is needed to decipher how terrain roughness affects biodiversity in other ecosystems. This research introduces novel tools for evaluating and conserving multi‐trophic biodiversity in forested ecosystems at the landscape scale. Recognizing the interconnectedness of these ecological variables is essential for better‐informed management policies and practices for biodiversity conservation and land management.

## Author Contributions

**Ayanna St. Rose:** conceptualization (equal), data curation (lead), formal analysis (lead), investigation (equal), methodology (equal), visualization (equal), writing – original draft (lead), writing – review and editing (supporting). **Kusum Naithani:** conceptualization (lead), formal analysis (supporting), funding acquisition (lead), investigation (lead), methodology (supporting), project administration (lead), resources (lead), supervision (lead), validation (equal), visualization (equal), writing – original draft (supporting), writing – review and editing (lead).

## Conflicts of Interest

The authors declare no conflicts of interest.

## Data Availability

Data used in this paper are available to download from the NEON data repository and the data analysis script is available to download from the GitHub repository at https://github.com/astrose/structure_diversity.
